# Pathogenesis of myasthenia gravis: update on disease types, models, and mechanisms

**DOI:** 10.12688/f1000research.8206.1

**Published:** 2016-06-27

**Authors:** William D. Phillips, Angela Vincent

**Affiliations:** 1 Physiology and Bosch Institute, University of Sydney, Anderson Stuart Bldg (F13), Sydney, 2006, Australia; 2 Neurosciences Group, Nuffield Department of Clinical Neurosciences, Weatherall Institute of Molecular Medicine, University of Oxford, Oxford, UK

**Keywords:** Myasthenia gravis, neuromuscular junction, immunoglobulin, AChR

## Abstract

Myasthenia gravis is an autoimmune disease of the neuromuscular junction (NMJ) caused by antibodies that attack components of the postsynaptic membrane, impair neuromuscular transmission, and lead to weakness and fatigue of skeletal muscle. This can be generalised or localised to certain muscle groups, and involvement of the bulbar and respiratory muscles can be life threatening. The pathogenesis of myasthenia gravis depends upon the target and isotype of the autoantibodies. Most cases are caused by immunoglobulin (Ig)G1 and IgG3 antibodies to the acetylcholine receptor (AChR). They produce complement-mediated damage and increase the rate of AChR turnover, both mechanisms causing loss of AChR from the postsynaptic membrane. The thymus gland is involved in many patients, and there are experimental and genetic approaches to understand the failure of immune tolerance to the AChR. In a proportion of those patients without AChR antibodies, antibodies to muscle-specific kinase (MuSK), or related proteins such as agrin and low-density lipoprotein receptor-related protein 4 (LRP4), are present. MuSK antibodies are predominantly IgG4 and cause disassembly of the neuromuscular junction by disrupting the physiological function of MuSK in synapse maintenance and adaptation. Here we discuss how knowledge of neuromuscular junction structure and function has fed into understanding the mechanisms of AChR and MuSK antibodies. Myasthenia gravis remains a paradigm for autoantibody-mediated conditions and these observations show how much there is still to learn about synaptic function and pathological mechanisms.

## Introduction

**Figure 1.**
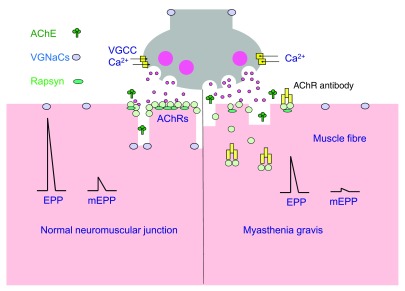
Assessing neuromuscular transmission. ( **A**) Healthy neuromuscular transmission. The nerve terminal can release the contents of each vesicle (quanta) of acetylcholine by exocytosis. Spontaneous release of single quanta of acetylcholine activates the intrinsic cation channels of acetylcholine receptors (AChRs) in the postsynaptic membrane to produce a small, transient depolarisation called a miniature endplate potential (mEPP). The nerve action potential opens voltage-gated calcium channels (VGCCs) and triggers exocytosis of many quanta of acetylcholine, simultaneously producing the (much larger) EPP. In healthy individuals, the amplitude of the EPP is more than enough to reach the threshold required to activate the postsynaptic voltage-gated sodium channels (VGNaCs) and generate a muscle action potential. ( **B**) The myasthenia gravis neuromuscular junction. AChR antibodies (mainly immunoglobulin [Ig]G1) activate complement, resulting in membrane attack complex-mediated damage to the post-junctional membrane architecture. The postsynaptic AChR numbers are depleted by divalent antibodies inducing AChR internalisation. The loss of AChRs results in smaller mEPP and EPP amplitudes. The EPP may not reach threshold, especially when the nerve is repetitively activated. Abbreviations: AChE, acetylcholinesterase

Myasthenia gravis (MG) is a paradigm autoantibody-mediated disease. Antibodies to the acetylcholine receptor (AChR) are found in 85% of patients with generalised muscle weakness and in 50% of those with purely ocular involvement ^1^. There is ample evidence from *in vitro* and *in vivo* approaches that these antibodies are pathogenic. AChR antibodies are typically of the immunoglobulin (Ig)G1 and IgG3 (human) subclasses, can lead to complement-mediated attack, and, being able to bind divalently to adjacent AChRs on the muscle surface, can also increase the rate of AChR internalisation (for a review of the earlier history of MG research, see 2). The resulting loss of AChRs at the neuromuscular junction (NMJ) impairs neuromuscular transmission (see Figure 1). This becomes clinically evident as fatigue and muscle weakness. In a minority of patients, however, the autoantibodies instead bind to muscle-specific kinase (MuSK). MuSK is a transmembrane tyrosine receptor kinase that is crucial for the development and maintenance of AChR clusters at the NMJ. These antibodies are clearly pathogenic, but the mechanisms are only recently beginning to be unravelled ^3^.

The pathogenic actions of autoantibodies at the level of the NMJ can be studied by a variety of techniques. Experiments on cultured muscle-like cells (TE671, C2C12 myotubes; outlined in 4) help define post-synaptic mechanisms in both AChR and MuSK antibody forms of the disease, but *in vivo* models are required to study the effects of the antibodies on the electrophysiology of neuromuscular transmission. A microelectrode can be used to record the membrane electrical potential of the muscle fibre near the NMJ. When the nerve is electrically stimulated, neuromuscular transmission can be detected as a brief rise in membrane potential, called the endplate potential (EPP ^5^). Spontaneous miniature EPPs (mEPPs), which are much smaller in amplitude than the (evoked) EPP, provide a measure of the response of the postsynaptic AChRs to release of a single synaptic vesicle-load (quantum) of acetylcholine. The quantal content refers to the number of vesicle-loads of acetylcholine released by the nerve terminal for each nerve impulse. Thus, the EPP amplitude is roughly equal to the mEPP amplitude multiplied by the quantal content.

Active immunisation of experimental animals against the affinity-purified AChR, passive transfer with rat- or mouse-derived mono-clonal antibodies specific for the AChR, or passive transfer of purified MG immunoglobulins containing high levels of AChR antibodies have all been informative ^6– 8^. Both passive transfer and active immunisation animal models result in a reduced postsynaptic response to acetylcholine (the neurotransmitter) measured as a reduction in the amplitude of the EPP and mEPPs ( Figure 1, normal on left and MG on right). As an animal becomes more severely affected, the EPP naturally becomes smaller and may not reach threshold for generation of the muscle action potential. A progressive failure of the action potential in a subset of myasthenic muscle fibres can be detected as a decrement in the compound muscle action potential (CMAP) amplitude during repetitive stimulation of the nerve ^5^.

Below, we provide an update and brief summary of the current understanding of these synaptic diseases including the pathogenic effects of AChR antibodies upon the motor endplate, and some less well-known aspects that have recently been reviewed in detail ^3, 9– 11^. This will be followed by recent approaches to begin to unravel the factors responsible for the failure of immune tolerance that leads to autoreactivity in MG. Finally, recent progress in our understanding of how MuSK autoantibodies cause NMJ failure will be discussed in detail.

## Mechanisms of AChR antibodies

AChR autoantibodies are mainly of the IgG1 and 3 subtypes, and so they are divalent and complement activating ^2^. Binding of these antibodies to AChRs results in activation of the classical complement pathway with assembly of the membrane attack complex (MAC). Calcium influx through the MAC causes local damage to the membrane, with release of AChR-containing membrane debris into the synaptic cleft ^11^. The damaged postsynaptic membrane shows a diminished response to acetylcholine, as measured electrophysiologically ( Figure 1) by reduced amplitudes of EPPs and mEPPs. Importantly, and not widely appreciated, the complement damage also causes a loss of voltage-gated sodium channels, which are located in the secondary folds, raising the threshold that the EPP must reach to trigger the muscle action potential ^12^. Bivalent AChR IgG can also cross-link adjacent AChRs, increasing the normally slow rate of internalisation and lysosomal degradation of the AChRs (normal half-life around 10 days in mice) and resulting in a loss of AChRs even in the absence of complement attack ( Figure 1) ^13^. Surprisingly, perhaps, most of the antibodies do not cause direct block of AChR function, although AChR block has been shown with a few individual patient sera ^14^.

There are many questions concerning the variability of muscle weakness between patients, and even within a patient. Some factors that could, in theory, contribute to this variability are the rate of diffusion of AChR antibodies from the serum into the very small synaptic cleft of each NMJ, the high number of the AChRs within this space that have to be targeted before a deficit in transmission occurs, and synaptic compensatory mechanisms that can be demonstrated in animal models. Regarding the latter, an increase in muscle AChR synthesis was found in passive IgG transfer experiments, and, similarly, increased mRNA for AChR subunits in biopsies from MG patients ^2^, and an increase in the quantal content of acetylcholine released from the nerve terminal during each nerve impulse ^15^. These adaptive responses would each tend to protect neuromuscular transmission from the pathogenic effects. The level of expression of tissue complement regulators could also influence the extent of NMJ damage ^11^. This is particularly important given that complement attack damages both the AChR-containing membrane (reducing sensitivity to acetylcholine) and the number of voltage-gated sodium channels (raising the threshold for the muscle action potential), as mentioned above ^12^. It seems likely that each of these modulating factors might differ between individuals and between muscles within an individual, explaining to some extent the variation in weakness and fatigue that is characteristic of all forms of MG.

## Recent approaches to investigating the failure of tolerance to AChR in MG

Most work in this area uses experimental models of MG, usually terminal experimental autoimmune MG (EAMG). This can be induced by active immunisation against purified AChR from electric organs of the marine ray, Torpedo, or electric eel, with adjuvants ^6, 16^. Torpedo AChR can be purified at high concentrations and in large amounts, making it highly suitable for EAMG induction. Unfortunately, only a proportion of the Torpedo AChR antibodies cross-react with mouse AChR to induce disease, and adjuvants are considered necessary to break tolerance. Thus, although EAMG results have helped to throw light on NMJ defects, the relevance of any immunological findings must be considered carefully. In a recent series of experiments, transgenic interleukin (IL)-17-null mice confirmed previous findings of the importance of T-helper cells that express the pro-inflammatory cytokine IL-17 ^17^. Since IL-17 is also expressed by other types of immune cells, the authors used adoptive transfer of CD4+ T cells from either wild-type or IL-17-null mice to repopulate IL-17-deficient mice before trying to induce EAMG. Host mice that were repopulated with wild-type CD4+ T cells developed antibodies against the injected Torpedo AChR and subsequently also developed autoantibodies against murine AChR. This was accompanied by myasthenic weakness. Host mice populated with IL-17 ^-/-^ CD4+ T cells developed similar levels of anti-Torpedo AChR but little anti-murine AChR and were resistant to EAMG ^17^. The authors could not detect any CD4+ T cells autoreactive for the murine AChR α-subunit. The findings therefore suggest that Th-17 cells do not play a role in the immune response to xenogeneic AChR but that they may facilitate the breaking of self-tolerance to the mouse (self) AChR. The cellular mechanisms involved remain to be defined.

Patients with AChR MG fall into three main categories: early onset MG (predominantly women <50), late onset MG (more frequently men over 50), and MG associated with thymoma. Since these early and late onset groups differ in their human leukocyte antigen (HLA) associations, and in their thymic pathology, but not their IgG AChR antibody characteristics ^18^, the distinctive clinical and aetiological characteristics suggest that the autoantibodies may arise via distinct pathogenic mechanisms operating within these different patient groupings ^19, 20^. The breaking of tolerance in early onset MG appears to involve the thymus, either primarily or secondarily, but human cellular studies have so far failed to identify the defects involved in antibody production.

Recent genome-wide association studies (GWAS) are making it possible to begin to dissect genetic predisposing factors for specific patient groups in MG. A GWAS of 649 early onset AChR MG patients from Northern Europe confirmed associations of AChR MG with the HLA class 1 region (specifically HLA-B*08) and with the ‘Protein Tyrosine Phosphatase, Non-Receptor Type 22’ (PTPN22) gene ^21^. The same study identified a novel association with the ‘TNFAIP3-interacting protein 1’ (TNIP1) gene. A more recent GWAS of 1032 white North American AChR patients revealed both similarities and differences between the early onset and late onset AChR MG patient groups ^22^. Both groups were associated with the HLA class 2 locus (albeit with distinct haplotypes) and with the ‘cytotoxic T-lymphocyte–associated protein 4’ gene (CTLA4, a T cell membrane protein previously implicated in autoimmune diseases). The late onset MG group specifically showed a strong association with ‘tumour necrosis factor receptor 4 superfamily, member 11a, NF-κB activator’ (TNFRSF11A), which encodes a protein involved in interactions between dendritic cells and T cells ^22^. These studies have begun to identify factors that might help to explain the early and late onset aetiologies. Additional, larger GWASs might allow dissection of distinct genes, alleles, and pathogenic mechanisms for different subsets of MG patients and could be particularly interesting with respect to the late onset MG patients who now represent a much higher proportion of the total ^23^.

## Mechanisms of MuSK antibodies

AChR MG is an immune-mediated disease with most of the effects dependent on the particular characteristics of the IgG antibodies. By contrast, MuSK MG appears to be principally a ‘pharmacological’ disease, where antibodies act to interfere directly with physiological mechanisms.

## MuSK IgG4 blocks MuSK signalling

**Figure 2.**
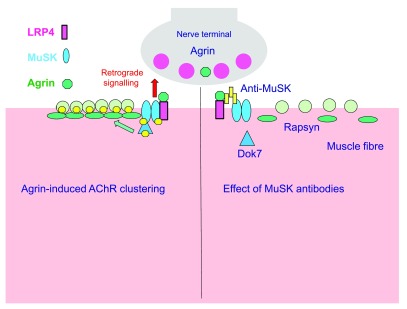
Disruption of postsynaptic differentiation pathway by muscle-specific kinase (MuSK) autoantibodies. ( **A**) Healthy MuSK-mediated postsynaptic differentiation pathway at the neuromuscular junction (NMJ). Neural agrin secreted by the motor nerve terminal binds to LRP4, low-density lipoprotein receptor-related protein 4 (LRP4), which causes the dimerisation of MuSK. MuSK dimerisation causes phosphorylation of MuSK and associated proteins of the MuSK pathway, including Dok7 and the acetylcholine receptor (AChR) β-subunit. Rapsyn is recruited to the phosphorylated AChRs, stabilising postsynaptic clusters of AChRs. ( **B**) Impaired postsynaptic differentiation in animal models of MuSK myasthenia gravis. MuSK autoantibodies are mainly of the immunoglobulin (Ig)G4 subclass. They block the assembly of the agrin-LRP4-MuSK complex. Interruption of MuSK kinase signalling leads to slow disassembly of the postsynaptic AChR clusters. A resultant decline in miniature endplate potential (mEPP) and EPP amplitude (not shown) results in failure of the muscle action potential and fatiguing weakness. Co-existing IgG1-3 antibodies, although lower concentration, may contribute but their pathogenic roles are not yet well defined. The compensatory presynaptic upregulation of quantal release found in AChR MG does not occur in MuSK MG.

Animal experiments show that MuSK IgG can cause MG. Mice that received repeated daily injections of patient IgG showed impaired neuromuscular transmission, with reductions in endplate AChR and in EPP amplitudes ^24– 30^. Similar changes to endplates were reported in mice, rats, and rabbits that were actively immunised with MuSK ^29, 31– 36^. Most of the MuSK in MG patient plasma is of the IgG4 subtype, with relatively low titres for IgG1-3 ^37, 38^. This is interesting because the IgG4 subclass lacks the complement-activating properties of IgG1 and is considered functionally monovalent ^39^, eliminating the two main pathogenic mechanisms of AChR MG. When the IgG4 and IgG1-3 fractions of MuSK patient IgG were separately injected into mice, the IgG4 fraction caused MG ^27^, while the IgG1-3 (but not with an equivalent amount of MuSK antibodies) did not. In the active immunisation model, complement-deficient mice that were immunised against MuSK developed MG that was even more severe than complement-sufficient strains ^35^. Thus, endplate damage by MuSK antibody does not appear to rely upon the classical immunopathology nor, because of lack of cross-linking, antigenic modulation mechanisms that drive AChR MG pathology. Furthermore, in the active and passive mouse models of AChR and MuSK MG, postsynaptic AChRs and the mEPPs were reduced to a similar extent but in the MuSK MG models there was no adaptive increase in the number of quanta of acetylcholine released by the nerve terminal ^27– 29, 35, 36^. Perhaps failure of presynaptic compensation explains why MuSK MG mice were weaker and MuSK MG patients are often more severely affected compared to AChR MG patients. The proposed effect of MuSK autoantibodies upon the mechanisms of postsynaptic differentiation and synaptic function is illustrated in Figure 2.

MuSK is found in the postsynaptic membrane of the NMJ, together with AChR ^40^. The protein tyrosine kinase function of MuSK is activated when agrin, a proteoglycan from the nerve terminal, binds to MuSK via the co-receptor ‘low-density lipoprotein receptor-related protein 4’ (LRP4) ^41– 44^. MG patient MuSK antibodies mainly bind the Ig-like regions in the MuSK ectodomain, thereby blocking assembly and activation of the agrin-LRP4-MuSK complex. This explains why agrin-induced AChR clustering in the C2C12 cell model was inhibited by incubation in MuSK MG sera and IgG preparations ^45– 47^. In mice injected with MuSK MG IgG, a reduction in postsynaptic tyrosine phosphorylation was associated with accelerated loss of AChRs from the postsynaptic AChR cluster ^30, 48^, culminating in failure of neuromuscular transmission ^28^. Thus, a combination of cell culture and mouse studies suggests that MuSK autoantibodies, which are mainly of the IgG4 type, block the natural activation of MuSK, leading to progressive loss of AChRs from the motor endplate and synaptic failure.

However, this may not be the whole story. Both the IgG4 and IgG1-3 fractions of MuSK MG plasma were able to inhibit agrin-induced AChR clustering when added to C2C12 muscle cell cultures. The intracellular protein Dok7 binds and stabilises the MuSK dimer, thereby enhancing MuSK’s tyrosine kinase activity ^49^. In a modified C2C12 model, AChR clustering was artificially induced by overexpressing Dok7. Despite the absence of agrin from this experimental system, both the IgG4 and IgG1-3 fractions still caused dispersal of the AChR clusters, suggesting that both IgG4 and IgG1-3 may affect MuSK independent of the interaction with LRP4 ^45^. Since IgG1-3 MuSK antibodies might also activate complement, it is too early to say that this IgG subclass plays no role. Conceivably, MuSK IgG1-3 antibodies might selectively affect certain muscle groups, for example those with especially high expression of MuSK ^50^, or where tissue complement regulators are deficient.

At healthy NMJs, there is a balance between clustering and cluster dispersal mechanisms. During embryonic development, and subsequently in mature muscle, MuSK functions to aggregate AChRs under the incoming motor nerve but, at the same time, acetylcholine released from the motor nerve terminal and acting upon these AChRs tends to dismantle AChR clusters ^51, 52^. It is thought that calcium influx through the AChR channel may be amplified by subsynaptic IP3 receptors ^53^, activating calcium-dependent proteases that then trigger the internalisation and degradation of AChRs, reducing AChR clusters. At healthy NMJs, synapse formation and synapse disassembly are balanced ^54, 55^. Impaired MuSK signalling in MuSK MG would disrupt this balance. This has clinical implications. Cholinesterase inhibitors, such as pyridostigmine, are a first-line treatment for MG. They prolong the activation of endplate AChRs and thereby restore the EPP amplitude. However, in MuSK MG patients, they are often not helpful or not tolerated ^56^. In the mouse passive IgG transfer model of MuSK MG (where MuSK signalling is inhibited), pyridostigmine was found to exacerbate endplate AChR loss and NMJ failure ^57^, probably by increasing and prolonging the dismantling action of acetylcholine on AChRs.

## Whittling down the ‘seronegative’ cases

A substantial fraction of MG patients reveal no detectable AChR or MuSK antibodies using the standard clinical radio-immunoprecipitation assays. Sensitive cell-based assays (CBAs) have recently shown that many of these ‘seronegative’ patients do indeed possess autoantibodies. These CBAs use fluorescently conjugated anti-human IgG to probe for patient antibodies binding to closely packed synaptic membrane proteins expressed on transfected cells. The CBAs can detect antibodies that recognise AChRs only when closely packed together, mimicking the close AChR packing at the endplate ^58, 59^. Close AChR packing may allow these antibodies to form stable divalent binding interactions, which are not possible in solution owing to the low concentration of AChRs. The AChR antibodies detected by CBA were mainly of the complement-fixing IgG1 subtype, similar to other AChR MG antibodies, and were able to passively transfer electrophysiological evidence of MG to mice ^58, 60^.

Other studies found that some double seronegative MG patients possessed LRP4 antibodies (mainly IgG1 and IgG2) ^61– 65^. Clearly antibodies to LRP4 could be pathogenic, and animals immunised against LRP4 demonstrate myasthenic weakness with impairment of neuromuscular transmission in mice ^66^, but the frequency of LRP4 antibodies has been variable. Antibodies to the secreted protein agrin, which is responsible for activating the LRP4/MuSK pathway, have been detected in small numbers of MG patients. However, most of the cases reported so far (10/12) also had antibodies to MuSK, LRP4, and/or AChR, and only two patients had no other antibodies detected ^67, 68^. The clinical and pathogenic significance of both LRP4 and agrin autoantibodies requires further investigation.

## Conclusions

Different subsets of MG patients develop autoantibodies with distinct target specificities, isotypes, and pathogenic mechanisms. Different pathogenic mechanisms then converge to cause loss of postsynaptic AChRs and increasing failure of neuromuscular transmission. This raises the need to investigate the immunological abnormalities specific to each of these categories of MG (as well as any common factors or pathways that might offer parsimonious therapeutic targets). The relative rarity of MuSK MG patients may make GWAS difficult, but the intriguing variation in the number of patients affected at different latitudes in the northern hemisphere (A. Vincent, unpublished data) raises the possibility of environmental factors contributing to disease aetiology. Mice actively immunised with MuSK generated a response characterised by IgG1 (which has characteristics similar to human IgG4), IL-4, and IL-10, analogous to the MuSK immunology found in MuSK MG patients ^32, 35, 69^, suggesting that there is something about the antigen itself that determines the immunological characteristics. Perhaps this mouse model will be useful for studying how and why IgG4 antibodies to MuSK arise.

Recent studies in MuSK MG have also focused attention on the molecular defences of the target organ: the NMJ. Local complement regulator proteins help protect the motor endplate from MAC-mediated damage in AChR MG ^70, 71^. Agrin/MuSK signalling provides a more general adaptive/protective response whenever there is a challenge to the function of the NMJ ^72^. Overexpression of MuSK or the intracellular MuSK-activator protein DOK7 protected muscles against NMJ impairment in transgenic mouse models of several neuromuscular diseases ^73, 74^. On the other hand, the NMJs of people carrying hypomorphic alleles for MuSK-pathway genes ^75^ might be more susceptible to AChR autoantibodies. Similarly, any hyper-activation of the postsynaptic IP3R1 receptor/calpain/caspase/CDK5 pathway ^52– 55^ conceivably might exacerbate the loss of postsynaptic AChR in AChR MG. These synapse-regulatory pathways offer potential targets for therapeutic interventions to ameliorate motor endplate damage in MG.

Some of the studies in animal models of MuSK MG reported changes in nerve terminal structure and/or presynaptic transmitter release ^24, 33, 35^. The presynaptic changes appear less robust than the postsynaptic changes. Nevertheless, the adaptive increase in presynaptic acetylcholine release that regularly occurs in models of AChR MG and in AChR MG patients ^15^ failed in models of MuSK MG. These findings suggest that MuSK signalling may help to mediate the presynaptic adaptive response. Ideally, some of the findings should be confirmed in patient muscle biopsies, particularly the most affected bulbar or facial muscles, but this remains a considerable challenge.

## Abbreviations

AChR, acetylcholine receptor; CBAs, cell-based assays; CMAP, compound muscle action potential; CTLA4, cytotoxic T-lymphocyte–associated protein 4; EPP, endplate potential; GWAS, genome-wide association study; HLA, human leukocyte antigen; Ig, immunoglobulin; IL-17, interleukin-17; LRP4, low-density lipoprotein receptor-related protein 4; MAC, membrane attack complex; MuSK, muscle-specific kinase; MG, myasthenia gravis; NMJ, neuromuscular junction; PTPN22, Protein Tyrosine Phosphatase, Non-Receptor Type 22; TNIP1, TNFAIP3-interacting protein 1; TNFRSF11A, tumour necrosis factor receptor 4 superfamily, member 11a, NF-κB activator.
